# Scarlet Fever

**Published:** 1870-06

**Authors:** 


### Scarlet Fever.

Sir William Jenner, Bart., M. D., F. R. S., Physician in Ordinary to her Majesty the Queen, discourses in the *Lancet*, as follows, with reference to scarlet fever: What is the treatment of uncomplicated scarlet fever, with trifling local specific process? For the poisoned condition of the system we have no remedy. There are those who say ammonia is the remedy; there are those who say that hydrochloric acid is the remedy; and so on with a variety of drugs. From experience we have no remedy for the general disease. We can only act upon the broadest general principles of calming the patient when excited; of stimulating him to keep the heart beating, when he is excessively weak; of cooling the surface when the heat is excessive. Give the patient pure fresh air to breathe. The *very* grave cases are hopeless from the first. For the mild cases little treatment is reqnired. A cool room, light clothes, unstimulating diet, a mild aperient, a chlorate of potash, which seems to allay the irritation or inflammation of the throat. Some give a little dilute mineral acid; sulphuric acid was all that used to be employed at the London Fever Hospital. He believes that the best remedy is the chlorate of potash drink. A drachm of the salt is put into a pint of barley water, and the patient is allowed to sip it down as he pleases.

By keeping the room well ventilated, any increase of fever is prevented, and the air is kept pure. The state of the urine, the skin, and the throat must be watched all the time. Dr. J. in concluding, lays great stress upon the following : changing the clothes of the patient, as well as noting the air in the room. The blankets of the bed should be changed frequently, as well as the body linen and sheets.—*Medical Gazette.*

